# Fusaric acid instigates the invasion of banana by *Fusarium oxysporum* f. sp. *cubense* TR4

**DOI:** 10.1111/nph.16193

**Published:** 2019-10-24

**Authors:** Siwen Liu, Jian Li, Yong Zhang, Na Liu, Altus Viljoen, Diane Mostert, Cunwu Zuo, Chunhua Hu, Fangcheng Bi, Huijun Gao, Ou Sheng, Guiming Deng, Qiaosong Yang, Tao Dong, Tongxin Dou, Ganjun Yi, Li‐Jun Ma, Chunyu Li

**Affiliations:** ^1^ Key Laboratory of South Subtropical Fruit Biology and Genetic Resource Utilization Ministry of Agriculture Key laboratory of Tropical and Subtropical Fruit Tree Research of Guangdong Province Institution of Fruit Tree Research Guangdong Academy of Agricultural Sciences Guangzhou 510640 Guangdong Province China; ^2^ College of Horticulture Shenyang Agricultural University Shenyang 110866 Liaoning Province China; ^3^ Institute of Biotechnology Zhejiang University Hangzhou 310058 China; ^4^ School of Life Sciences Sun Yat‐sen University Guangzhou 510275 China; ^5^ Department of Biochemistry and Molecular Biology University of Massachusetts Amherst MA 01003 USA; ^6^ Department of Plant Pathology University of Stellenbosch Private Bag X1 Matieland 7602 South Africa

**Keywords:** banana, fusaric acid, *Fusarium oxysporum* f. sp. *cubense *TR4, reactive oxygen species, virulence

## Abstract

- Fusaric acid (FSA) is a phytotoxin produced by several *Fusarium* species and has been associated with plant disease development, although its role is still not well understood.
- Mutation of key genes in the FSA biosynthetic gene (*FUB*) cluster in *Fusarium oxysporum* f. sp. *cubense* tropical race 4 (*Foc* TR4) reduced the FSA production, and resulted in decreased disease symptoms and reduced fungal biomass in the host banana plants.
- When pretreated with FSA, both banana leaves and pseudostems exhibited increased sensitivity to *Foc* TR4 invasion. Banana embryogenic cell suspensions (ECSs) treated with FSA exhibited a lower rate of O_2_ uptake, loss of mitochondrial membrane potential, increased reactive oxygen species (ROS) accumulation, and greater nuclear condensation and cell death. Consistently, transcriptomic analysis of FSA‐treated ECSs showed that FSA may induce plant cell death through regulating the expression of genes involved in mitochondrial functions.
- The results herein demonstrated that the FSA from *Foc* TR4 functions as a positive virulence factor and acts at the early stage of the disease development before the appearance of the fungal hyphae in the infected tissues.

## Introduction

Banana (*Musa* spp.) is one of the most important economic crops for many Asian and African countries. *Fusarium oxysporum* f. sp. *cubense* (*Foc*) causes Fusarium wilt (Panama disease), one of the most destructive diseases of banana trees (Moore *et al*., 1993). Based on the different susceptibilities from different host plants, *Foc* is divided into four different physiological races. *Foc* race 1 causes diseases in banana cultivars ‘Gros Michel’ (AAA group) and ‘Silk’ (Waite & Stover, 1960). During the 20^th^ Century, the banana industry that was almost exclusively based on the ‘Gros Michel’ cultivar was nearly completely destroyed by the *Foc* race 1 infection, until the race 1 resistant cultivar ‘Cavendish’ (AAA) appeared and became the dominant cultivar for the industry. *Foc* race 2 affects the hybrid triploid cultivar ‘Bluggoe’ (ABB) and other cultivars closely related to ‘Bluggoe’ (Ploetz, 2015). *Foc* race 3 is only virulent on the ornamental *Heliconia* spp. but does not affect the *Musa* spp. Therefore, it is not considered *cubense* anymore (Ploetz *et al*., 2015b). *Foc* race 4, however, has a very broad host range including the ‘Cavendish’ and all the cultivars susceptible to race 1 and race 2. *Foc* race 4 is divided into the *Foc* ‘subtropical’ race 4 (STR4) and the *Foc* ‘tropical’ race 4 (TR4) strains that affect the Cavendish bananas in the subtropics and tropics, respectively. Between these two strains, *Foc* TR4 is the more virulent one as it can infect Cavendish bananas under both stressed and nonstressed conditions (Li *et al*., 2013). A lack of effective quarantine measures has resulted in *Foc* TR4 spreading from the Asia‐Pacific region, where it was restricted for more than two decades, to the Middle East and countries of the Greater Mekong Subregion (Garcia‐Bastidas *et al*., 2014; Ploetz *et al*., 2015a; Zheng *et al*., 2018). With the exceptions of a few resistant/tolerant varieties, no effective measures to manage the disease have been developed for this pathogen.

*Foc* is a soilborne pathogen that usually infects the roots first. Conidia and hyphae can be detected on root surfaces as early as 2 d post‐inoculation (dpi) (Li *et al*., 2017). Using the green fluorescent protein‐expressing *Foc*, it has been demonstrated that the pathogen moves from the soil into the roots and rhizome (Li *et al*., 2011, 2017). Furthermore, hyphae seem to be able to penetrate into the root xylem 10 dpi and spread into the rhizome and pseudostem xylem after another several days (Xiao *et al*., 2013; Warman & Aitken, 2018). The movement of *Foc* into the vascular bundle seems to be important for the banana wilting development, because the wilt is caused mainly by the blocking of the transport of nutrients and water (Li *et al*., 2017). In addition, *Foc* is able to reach into the outer sheaths of senesced leaves of the pseudostem before the appearance of the more visible symptoms (Warman & Aitken, 2018).

*Foc* is a hemibiotrophic pathogen and utilizes an array of virulent factors to help its infection of the host plants. A few fungal mechanisms and molecules contribute to the pathogenicity of *Foc* TR4. These include the effector proteins encoded by the *SIX* (secreted into xylem) genes (Widinugraheni *et al*., 2018) and the phytotoxic secondary metabolites fusaric acid (FSA) (Li *et al*., 2013b; Ding *et al*., 2018) and beauvericin (BEA) (Li *et al*., 2013b). FSA is a phytotoxin produced by several *Fusarium* species and other fungal pathogens (Fakhouri *et al*., 2003), and known to have strong phytotoxicity to both animal and plant cells. In maize roots, FSA was shown to impair the respiration activities in mitochondria (Telles‐Pupulin *et al*., 1996). In tomato plants, FSA showed a strong phytotoxic effect, potentially functioning as a chelating agent of copper, iron or zinc (Lopez‐Diaz *et al*., 2018). At lower concentration, FSA was able to induce the plant defense response involving the production of reactive oxygen species (ROS) in *Arabidopsis* (Bouizgarne *et al*., 2006a) and tomato (Singh *et al*., 2017). Likewise, at low doses, FSA was able to induce the programmed cell death and symptoms of apoptosis responses including the production of H_2_O_2_ and DNA fragmentation in saffron (*Crocus sativus*) (Samadi & Shahsavan Behboodi, 2006) and tobacco suspension cells (Jiao *et al*., 2013).

There seems to be a tight correlation between the presence of FSA and the appearance of banana wilt disease symptoms, and FSA may damage the host cell membranes leading to leaf water loss and electrolyte leakage (Dong *et al*., 2012). However, other detailed molecular functions of FSA in the development of banana wilt remain unclear. The toxicity of FSA to banana protoplasts, pseudostems and plantlets have been demonstrated, as has its accumulation in the host plants (Li *et al*., 2013b). The present study combines cell biology, reverse genetics, RNA‐Seq and disease assays to provide a comprehensive analysis of FSA function at the *Foc* TR4–banana interface. The data revealnovel characteristics of FSA and demonstrated that FSA could act as a pioneer molecule to disturb mitochondrial functions and induce cell death, thus preparing the host for the upward invasion of *Foc* TR4.

## Materials and Methods

### Fungal strains and media

*Fusarium oxysporum* f. sp. *cubense* (*Foc*) ‘tropical’ race 4 (TR4) strain II5 (VCG01213) was used as the wild‐type (WT) for fungal transformation, targeted gene disruption and complementation experiments. All *Foc* strains were cultivated on potato dextrose agar (PDA) at 28°C.

### Phylogeny analysis

The genomes of all of the *Fusarium* species were downloaded from the given link: http://www.broadinstitute.org/annotation/genome/fusarium_group/MultiDownloads.html. PhyML 3.1 (Guindon *et al*., 2010) was used to generate the maximum‐likelihood trees. mrbayes v.3.2 was used for tree construction by Bayesian inference (Guindon *et al*., 2010). The SH test (Halilovič *et al*., 2011) was implemented in raxml v.8.2.12 (Stamatakis, 2014) to determine whether there was a significant difference in the tree topologies supported by different genes.

### Pathogenicity assay, confocal microscopic observation of infection and fungal biomass estimation

The TR4‐susceptible ‘Cavendish’ banana (AAA) cv ‘Brazilian’ plantlets with six to seven leaves (*c*. 25 cm height) were inoculated with *Foc* TR4 isolates at a concentration of 1000 conidia g^−1^ of soil.

For microscopic examination, banana roots and pseudostems were prepared and observed under a laser confocal microscope (LSM 710; Carl Zeiss, Oberkochen, Germany) equipped with filter blocks with spectral properties matching those of green fluorescent protein (GFP) (488 nm) and root autofluorescence (543 and 595 nm) (Li *et al*., 2013a). To determine the fungal hyphae and spores inside the pseudostem, an observation site at 5 cm above the corm of each plant was chosen, and the transverse slices were cut and used for observing the appearance of fluorescent fungi.

For pathogenicity assessment, the disease severity of each tested plant was evaluated. The disease index was ranked as: 0, no symptoms; 1, some brown spots in the inner rhizome; 2, < 25% of the inner rhizome show browning; 3, up to 3/4 of the inner rhizome show browning; and 4, entire inner rhizome and pseudostem are dark brown, dead).

Fungal biomass was determined using the method described previously (Thatcher *et al*., 2009). Quantitative real‐time PCR (qRT‐PCR) was performed using an CFX96 real‐time PCR system (Bio‐Rad) in combination with the SYBR Premix Ex Taq kit (TaKaRa, Kusatsu, Japan). Elongation factor 1‐α (*EF1*α) from *Foc* TR4 was used to qualify fungal colonization and banana actin gene (*MusaActin*) was used as an endogenous plant control.

### Analysis of FSA

The FSA content was determined via liquid chromatography‐tandem mass spectrometry (LC‐MS/MS) analysis as described previously (Li *et al*., 2013b). The amount of ergosterol was used as the internal control in each *Foc* sample incubated on PDA plates. Ergosterol was extracted and analyzed as described previously (Guo *et al*., 2015). The sap was extracted from the 10‐cm length of pseudostem above the observation site, and FSA content was calculated basing on the ergosterol content.

### qRT‐PCR analysis

Total RNA samples were extracted using an RNA Out Kit (Tiandz, Beijing, China) following the manufacturer's instructions. For each sample, 1 μg of RNA was reverse‐transcribed into cDNA using a PrimeScript RT reagent kit with gDNA Eraser (Takara). The qRT‐PCR mixtures (each contained 10 μl of 2 × SYBR Premix Ex Taq buffer, 1 μg of synthesized cDNA and 10 μmol of each gene‐specific primer in a final volume of 20 μl) were prepared using the SYBR Premix Ex Taq kit (Takara). The qRT‐PCR was performed with three technical replicates in a CFX96 real‐time PCR system (Bio‐Rad). *FocEF1*α (*Foc*) and *MusaActin* (banana) were used as internal controls to normalize the data. The relative expressions of genes were calculated using the 2^−ΔΔCT^ method. The gene‐specific primers used in the qRT‐PCR analysis are listed in Supporting Information Table S1. The abbreviations of genes are shown in the Table S2.

### RNA‐Seq analysis

Total RNA was extracted from Cavendish banana embryogenic cell suspensions (ECSs) treated with FSA for 0, 6 and 24 h using an Illumina standard library preparation kit. Each treatment had three biological replicates. RNA quality and integrity were confirmed with a minimum RNA integrity number (RIN) value of 7. RNA‐Seq reads were generated via Illumina HiSeq 2000 platform at Beijing Novogene Biotech Co, Ltd (Beijing, China). fastqc was used to remove poor‐quality bases and low‐quality reads as well as sequencing adaptors used in the process. The trimmed reads were mapped to banana genome (*Musa acuminate* DH Pahang v2) using *SOAP* aligner allowing up to two base mismatches per read. The RPKM (reads per kb per million reads) was calculated to reflect the expression level of banana transcripts. For gene expression comparison, differentially expressed genes were identified with the criterion of the absolute log_2_ (RPKM fold‐change) value ≥ 1 and false discovery rate ≤ 0.05.

### Protein extraction and Western blotting

The protein isolation and Western blot analysis of banana ECSs were performed as previously (Gao *et al*., 2017). Briefly, total proteins of Cavendish banana ECS lysates were prepared in ice‐cold phosphate‐buffered saline (PBS) (100 mM, pH 7.5) containing 1 mM EDTA, 100 μg ml^−1^ PMSF, 0.1% Triton X‐100 and protease/phosphatase inhibitor cocktails (Roche). For Western blotting, protein samples (20 μg each) were resolved via sodium dodecyl sulfate polyacrylamide gel electrophoresis (SDS‐PAGE) and transferred to a polyvinylidene difluoride (PVDF) membrane. Protein detection via Western blotting used the anti‐HSP70 and anti‐AtpB (1 : 10 000) antibodies (Sangon Biotech, Shanghai, China).

### Measurement of the mitochondrial membrane potential and ATP

Mitochondrial membrane potential (Δ*Ψm*) was analyzed via JC‐1 staining (Yao *et al*., 2002). Samples were first incubated with the JC‐1 stain for 30 min at room tempreature in the dark, then washed three times with PBS. The ATP content was determined using an ATP assay kit (MAK190) according to the manufacturer's instructions (Sigma).

### Generation of deletion mutants

The primers used are listed in Table S1. The PCR products were transformed into the protoplasts of the WT strain II5 via polyethylene glycol (PEG)‐mediated protoplast transformation (Gu *et al*., 2015). Hygromycin B (Calbiochem, La Jolla, CA, USA) was used at the final concentration of 150 mg ml^−1^ for transformant selection. Putative deletion mutants were identified through PCR assays with the primer pairs listed in Table S1 and further confirmed by Southern hybridizations (Fig. S1). Complementation of these mutants was achieved by transformation with complementary fragments containing the full‐length genes and the corresponding native promoter regions. The fragments were amplified via PCR with specific primers (Table S1) and inserted into pYF11 (bleomycin resistance) to complement the mutant strains.

### Protoplast isolation and treatment

The ECSs of Cavendish banana (AAA) cv ‘Brazilian’ were used as donor materials to isolate protoplasts 7 d after the final subculture. The cells were centrifuged at 600 ***g*** for 5 min, and the culture medium was replaced with 10 ml of an enzyme solution containing 1.5% (w/v) cellulase RS (Yakult Honsha Co., Tokyo, Japan), 0.15% (w/v) pectolyase Y 23 (Kyowa Chemical Products Co., Osaka, Japan), 204 mM KCl and 67 mM CaCl_2_ (pH 5.6). The enzyme–cell suspension mixture was incubated on a shaker at 80 rpm for 3 h at 28°C. The protoplasts were washed twice with 204 mM KCl and 67 mM CaCl_2_. The cells were collected by centrifugation at 600 ***g*** for 5 min each time.

### Measurement of respiration in banana ECS protoplasts

The impact of FSA on cellular respiration was determined by measuring the oxygen consumption rate (OCR) of the protoplasts using an XF96e Flux Analyzer (Seahorse Biosciences, North Billerica, MA, USA) in real time. Thirty minutes before the assay, the growth media were replaced with XF assay media (10 mM glucose, 4 mM l‐glutamine and 2 mM sodium pyruvate, pH 7.4), and protoplasts were seeded onto poly‐d‐lysine‐coated XF 96‐well cell culture microplates (Seahorse Bioscience) at a density of 1 × 10^5^ cells per well. The optimal concentrations of the pharmacological agents (oligomycin (8 μM), phenylhydrazone (FCCP; 6 μM) and antimycin A (5 μM)) were determined by titrations before the FSA treatment assay. During the FSA treatment assay, four compounds were serially injected, shifting the bioenergetic profile of the cells. Basal respiration is primarily controlled by ATP synthase and proton leakage. Injection of the ATP coupler oligomycin blocks ATP synthase and residual respiration due to a proton leak, resulting in a significant decrease in OCR. FCCP uncouples oxygen consumption from ATP production, which induces high artificial proton conductance into the membrane. A candidate uncoupler or the respiration inhibitor FSA was added after FCCP treatment. Finally, the electron transport complex III inhibitor antimycin A was added to inhibit total mitochondrial respiration. The results were averages recorded from four replicate wells for each treatment group.

### Subcellular localization of H_2_O_2_

The subcellular localization of H_2_O_2_ was determined based on the generation of cerium perhydroxides as described previously (Yao *et al*., 2002). Ultrathin sections of banana leaves (90 nm) were generated on a microtome (Leica EM UC6, Wetzlar, Germany) using a diamond knife (Diatome) and examined without staining. The images were recorded using a transmission electron microscope (JEM‐1400; Jeol Peabody, MA, USA) at an accelerating voltage of 120 KV.

### Detection of reactive oxygen species (ROS) in banana ECS protoplasts

Protoplasts from 7‐d‐old banana ECSs were treated with 0.1% MEOH (CK) or 300 μM FSA, or co‐incubated with the ROS scavenger *N*‐acetylcysteine (NAC; (5 μg ml^−1^) or the NADPH oxidase inhibitor (DPI), for 5 min. Samples were then double stained with CM‐H_2_DCFDA (to detect ROS) and CMXRos (mitochondrial marker). Images were taken using a confocal laser scanning microscopy (LSM 710).

### Alkaline comet assay

An alkaline comet assay reagent kit (Trevigen, Gaithersburg, MD, USA) was used according to the manufacturer's instructions to evaluate DNA damage in the banana protoplasts, including single‐strand breaks (SSBs), double‐strand breaks (DSBs) and alkali labile sites (ALSs). FSA (300 μM) was added to the assays in 24 and 48 h reactions. Comet parameters, including the tail DNA (%), tail moment (arbitrary units, AU) and tail length (μm), were assessed with a comet assay image analysis system (Comet Assay Software Project, CASP Lab, Poland).

### Histochemical assays

The presence of ROS in the leaf samples was determined as described previously (Bi *et al*., 2014). The 3'‐diaminobenzidine (DAB)‐stained leaves were observed under a stereomicroscope (SteREO Lumar.V12; Carl Zeiss) equipped with a CCD camera (AxioCamMRc; Carl Zeiss). The data were analyzed using imagej software as described previously (Taheri & Kakooee, 2017). The DAB staining was recorded as the area of staining divided by the total leaf area. Three independent tests were conducted.

Trypan blue staining was performed to visualize cell death as described previously (Houterman *et al*., 2007). Leaves were boiled for 5 min in a 1 : 1 mixture of ethanol and 0.33 mg ml^−1^ trypan blue dissolved in lactophenol, then destained overnight in 2.5 g ml^−1^ chloral hydrate in water. The stained leaves were examined under a microscope (Carl Zeiss) with a CCD camera (AxioCamHRc; Carl Zeiss). The experiments were repeated at least three times.

### Pathogen assay after altering the ROS concentration

In order to alter the level of oxidative burst, a ROS‐generating system or a ROS‐scavenger was used as described previously (Taheri & Kakooee, 2017). Leaves were treated with either glucose + glucose oxidase (G + GO; 2.5 mM, 25 units ml^−1^) or xanthine + xanthine oxidase (X + XO; 0.5 mM, 0.05 units ml^−1^) in 10 mM PBS (pH 7.0) for 3 h to generate H_2_O_2_ or O_2_, respectively. Two hours after each treatment, the discs were inoculated with the *Foc* spore suspension (100 spores ml^−1^ concentration). In the antioxidant assays, the leaf discs were treated for 1 h with 10 μM diphenyleneiodonium chloride (DPI) to inhibit the NADPH oxidase activities, similar to the procedure used for the FSA treatment. The leaf inoculation was carried out using a spore suspension of the pathogen as described previously (Govrin & Levine, 2000). Disease progression was evaluated at 5 d post‐inoculation (dpi) by measuring the lesion length using the leica las software (Wetzlar, Germany). The experiments were repeated at least three times.

## Results

### Identification of the *FUB* cluster in *Foc* TR4

**Figure 1:**
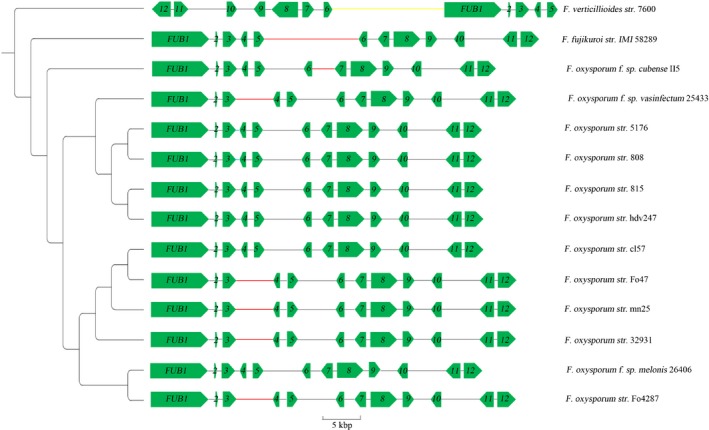
Phylogeny tree and structural variations of the *FUB* (Fusaric acid biosynthetic) gene clusters from *Fusarium* species. The phylogenetic tree was constructed based on the concatenated sequences of the 12 *FUB* gene coding sequences. All branches on the phylogenetic tree had 100 percent bootstrap support rate by maximum‐likelihood method and 100% posterior probability by Bayesian inference. The 12 *FUB* genes (denoted as *FUB1* and *2–12* in the graph) are represented by green arrows. Structural variants with minor allelic frequency were denoted using bold lines in yellow (translocation), red (insertion > 50 bp) and blue (deletion > 50 bp).

It was reported that there are 12 genes in the Fusaric acid biosynthetic (*FUB*) cluster in *F. verticillioides*,* F. fujikuroi* and strains of *F. oxysporum* (Brown *et al*., 2015). The genomes of all 12 *F. oxysporum* species were downloaded from the Fusarium Comparative Database (FCD) at the Broad Institute ( http://www.broadinstitute.org/annotation/genome/fusarium_group/MultiDownloads.html), together with the genomes of *F. verticillioides* str. 7600 and *F. fujikuroi* str. IMI 58289 as comparison controls (Fig. 1). The *FUB* clusters of genes were identified from all of these strains based on comparative genomic analysis. Except for *F. verticillioides* str. 7600, all of the other strains showed similar arrangements of the 12 *FUB* genes (Fig. 1). Gene synteny of these genes in different *F. oxysporum* strains were generally consistent, and several observed structural variations were all located in intergenic regions (Fig. 1). Discrepancy between the intergenic region patterns of *FUB3* and *FUB4*, and the molecular phylogeny based on the *FUB* gene clusters were observed. These observations suggest that horizontal gene transfer events may have happened in the ancestors of at least three *F. oxysporum* isolates (*F. oxysporum* str. 25433, *F. oxysporum* str. Cl57,and *F. oxysporum* str. 26406 or *F. oxysporum* str. 4287). A specific replacement was observed on the *FUB6*/*FUB7* intergenic region in *F. oxysporum* f. sp. *cubense* II5. This region is 2865 bp and showed no sequence similarity to the same intergenic region that was homologous in other *F. oxysporum* isolates (1281–1320 bp in length). From a blast search against other *Fusarium* genomes, no homologous sequence of the II5 *FUB6*/*FUB7* intergenic region could be identified in any other *F. oxysporum* isolate. However, a 148‐bp region of the sequence showed 93% similarity to a sequence of similar length that presents in multiple locations in the genome of *Fusarium poae*, suggesting that the region in II5 could have been introgressed from a distant *Fusarium* species.

### *FUB* genes are involved in the biosynthesis of FSA

In order to investigate the functions of the *FUB* genes in *Foc* TR4, *Foc* homologs *FUB1*,* FUB2*,* FUB3*,* FUB4*,* FUB5* and *FUB10* were deleted individually, and the mutants were confirmed by PCR diagnosis and Southern hybridization analysis (Table S1; Fig. S1). In addition to the six single‐gene deletion mutants (*fub1, fub2, fub3, fub4, fub5* and *fub10*), their corresponding complementation mutants also were obtained (*fub1*‐C*, fub2*‐C*, fub3*‐C*, fub4*‐C*, fub5*‐C and *fub10*‐C) (Fig. S1), which also were identified by PCR with specific primers (Table S1). The expressions of these six genes in each deletion mutant and the wild‐type (WT) progenitor strain II5 were examined using qRT‐PCR over the course of 9 d of culturing (Fig. S2). Their expressions were not detected in the corresponding mutants, indicating that the disruption of each *FUB* gene resulted in a null mutant. In WT, the transcript levels of *FUB*10 showed a sustained increase over the analyzed time‐course (Fig. S2). The other *FUB* genes did not present a regular expression pattern, but they were all upregulated in WT from Day 3 to Day 6 (Fig. S2).

**Figure 2:**
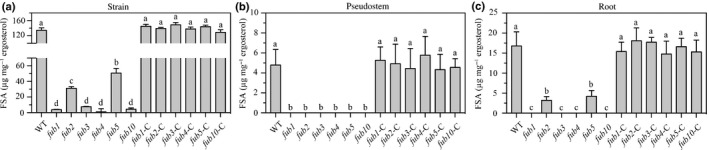
Fusaric acid (FSA) concentrations (μg^−1^ fungal ergosterol) of the wild‐type (WT) and *Fusaric acid biosynthetic* (*fub*) mutant strains: (a) in cultures; (b) in the sap of pseudostems of inoculated banana plants; and (c) in the roots of inoculated banana plants. Data are means ± SD from three independent experiments and different letters above the columns indicate the significant difference (*P* < 0.05) between WT and mutant strains at the same time point.

All *FUB* mutants of the *Foc* isolate and their corresponding complementation strains were tested for the productions of FSA both in cultures and within banana plants. According to the LC‐MS/MS analysis of the liquid media cultured with the mutants, FSA production was severely impaired in *fub1* (3%), *fub3* (5%), *fub4* (1%) and *fub10* (4%). Mutants *fub2* and *fub5* still showed 30% and 50% of FSA relative to the WT, respectively (Fig. 2a), confirming that the proteins encoded by these genes are critical enzymes involved in the FSA production in *Foc* TR4. FSA concentrations in sap and roots also were measured. The FSA concentration in sap of banana plantlets inoculated with *FUB* mutants was below the threshold (4.2 ng mg^−1^ ergosterol) of the LC‐MS/MS detection (Fig. 2b). Similar results were obtained in the root samples inoculated with the *fub1*,* fub3*,* fub4* and *fub10* mutants, whereas very low FSA concentrations were detected for the root samples inoculated with *fub2* and *fub5* (Fig. 2c). All complementation of the deletion strains with corresponding WT gene fully restored their defects in FSA production (Fig. 2). In addition, no difference in morphology and growth rate was found for any of the mutants on potato dextrose agar (PDA) (Fig. S1), indicating that the absence or inhibition of the production of FSA does not affect the vegetative growth of *Foc* TR4 strains.

### FSA contributes to the virulence of *Foc* TR4

**Figure 3:**
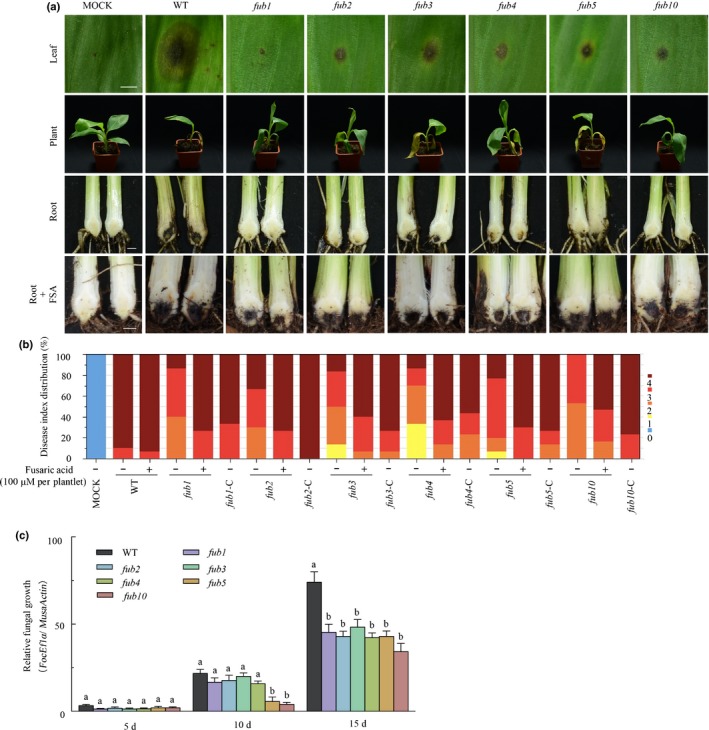
Targeted disruption of *Fusaric acid biosynthetic* (*FUB*) genes impairs the virulence of *Fusarium oxysporum* f. sp. *cubense* tropical race 4 (*Foc* TR4) on banana plants. (a) Disease phenotype. Bars: leaf, 0.2 cm; root, 1 cm. (b) Disease index of inoculated plants. Disease severity was assessed on a four‐scale rating standard. (c) *In planta* fungal growth in roots of the inoculated plants, evaluated by quantitative PCR analysis of *FocEF1*α and banana *actin* genes and shown as ratios of *FocEF1*α* : MusaActin*. Data presented in (c) are the means ± SD from three independent experiments and different letters above the columns indicate the significant difference (*P* < 0.01) between wild‐type (WT) and mutant strains at the same time point.

In order to investigate the correlation between the biosynthesis of FSA and *Foc* TR4 virulence during host infection, the relationship between the amount of FSA produced by these *fub* mutants and their pathogenicity on banana plantlets was evaluated**.** All *Foc* strains, including the WT (*Foc* TR4 II5), the *fub* mutants and their complementation mutants, were able to infect the Cavendish banana plantlets when inoculated with 1000 conidia g^−1^ of soil, resulting in wilting symptoms on the banana plantlets. However, it took much longer for disease symptoms to develop for the mutants (*c*. 51 dpi) compared to the WT and the complementation strains (*c*. 37 dpi) (Fig. 3a), and the disease indices (DIs) of the *fub* mutants were much lower than those of the WT and complementation strains (Fig. 3b). The function of FSA in disease development was further tested on the surface of Cavendish banana plantlet leaves. The results showed that all *Foc*‐inoculated leaves exhibited necrotic lesions on the leaf surfaces. However, compared to the WT and the complementation strains, the development of lesions caused by the *fub* mutant strains were delayed, and the sizes of the lesions were relatively smaller (Fig. 3a). To determine whether the targeted disruption of key genes in the FSA biosynthesis pathway affected fungal growth *in planta*, the fungal biomass in the inoculated banana roots was quantified. The fungal biomass in root of the WT strain‐inoculated plants increased during 5–15 dpi. However, the fungal biomass in root of *fub1*‐, *fub2*‐, *fub3*‐, *fub4*‐, *fub5*‐ and *fub10*‐inoculated plants were significantly reduced by 1.6‐, 1.7‐, 1.5‐, 1.7‐, 1.7‐ and 2.1‐fold (respectively) at *P* < 0.01 when compared with that of WT‐inoculated plants (Fig. 3c). These data indicate that the targeted deletion of *FUB* genes to inhibit FSA production reduced the virulence of *Foc* TR4 in Cavendish banana and lessened the ability of the pathogens to grow within banana root tissues, further demonstrating that FSA is required for the virulence of *Foc* TR4 in banana plantlets.

In order to determine whether FSA is responsible for invasion defects of *fub* deletion mutants, FSA (100 μM per plantlet, which is similar to the concentration of endogenous FSA) was added onto *fub* mutant‐inoculated banana plantlets. Notably, the ability of each *fub* mutant to invade banana was restored by FSA at 40 dpi and the addition of FSA even enhanced banana infection with the WT *Foc* TR4 (Fig. 3a,b).

### FSA diffuses ahead of the invasion of *Foc* TR4 in banana pseudostems

**Figure 4:**
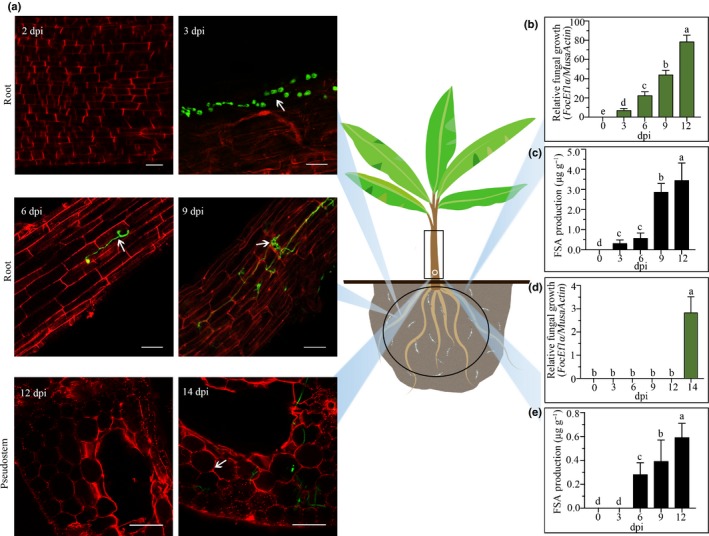
Fusaric acid (FSA) spreads ahead of the invasion of *Fusarium oxysporum* f. sp. *cubense* tropical race 4 (*Foc* TR4) inside banana plant. (a) Detection of GFP‐tagged *Foc* TR4 (green, arrows) by Laser Confocal Microscope in Cavendish banana roots from 2 to 9 d post‐inoculation (dpi) and in pseudostems at 12 and 14 dpi. Bars: root, 50 μm; pseudostem, 200 μm). (b–e) The relative fungal growth (b, d) and FSA production (c, e) from Day 0 to 12 dpi in roots (d, e) and pseudostems (b, c). Data presented in (b) to (e) are the means ± SD from three independent experiments and different letters above the columns indicate the significant difference (*P* < 0.05) among the different time points.

The TR4‐susceptible Cavendish banana variety Brazilian (AAA) plantlets with six to seven leaves (*c*. 25 cm height) were inoculated with GFP‐tagged *Foc* TR4 (GFP‐TR4) isolates at a concentration of 1000 conidia g^−1^ soil. In the root system, no fungal spores or hyphae were found on the root surface at 2 dpi (Fig. 4a), but a few spores were observed attached to the epidermal cell surfaces of the lateral roots at 3 dpi (Fig. 4a). At 6 dpi, the fungus seemed to have established on the root system, because GFP‐TR4 began to expand its hyphal network by growing into the intercellular spaces along the junctions of the root epidermal cells (Fig. 4a). By 9 dpi, GFP‐TR4 had established infections within the plant cells, with the fungal hyphae proceeding rapidly and the concomitant production of a network of branching hyphae (Fig. 4a). However, the fungus growth and infection seemed much slower in the pseudostems compared to what was observed in the roots. The presence of GFP‐TR4 hyphal network was still not detected in the pseudostems by 12 dpi, although it was detected at 14 dpi.

During the course of infection, the growth of GFP‐TR4 in the inoculated plants was monitored by qPCR to measure the cell proliferation (Fig. 4b,d) and by LC‐MS/MS to measure the FSA concentration (Fig. 4c,e). The pathogen multiplied in the roots as indicated by the qPCR results (Fig. 4b) and the FSA content increased correspondingly in a similar course (Fig. 4c). However, the accumulation of FSA seemed to precede the pathogen multiplication in the pseudostem (10 cm above the observation site). As the qPCR results showed, GFP‐TR4 was detectable in the pseudostem only at 14 dpi (Fig. 4d), but FSA was detected in the xylem sap of the pseudostem as early as 6 dpi (Fig. 4e). Therefore, it seems that FSA may be able to diffuse with sap through the vascular tissues ahead of the arrival and colonization of the pathogens.

### Pretreatment of pseudostems with FSA promotes *Foc* TR4 invasion

**Figure 5:**
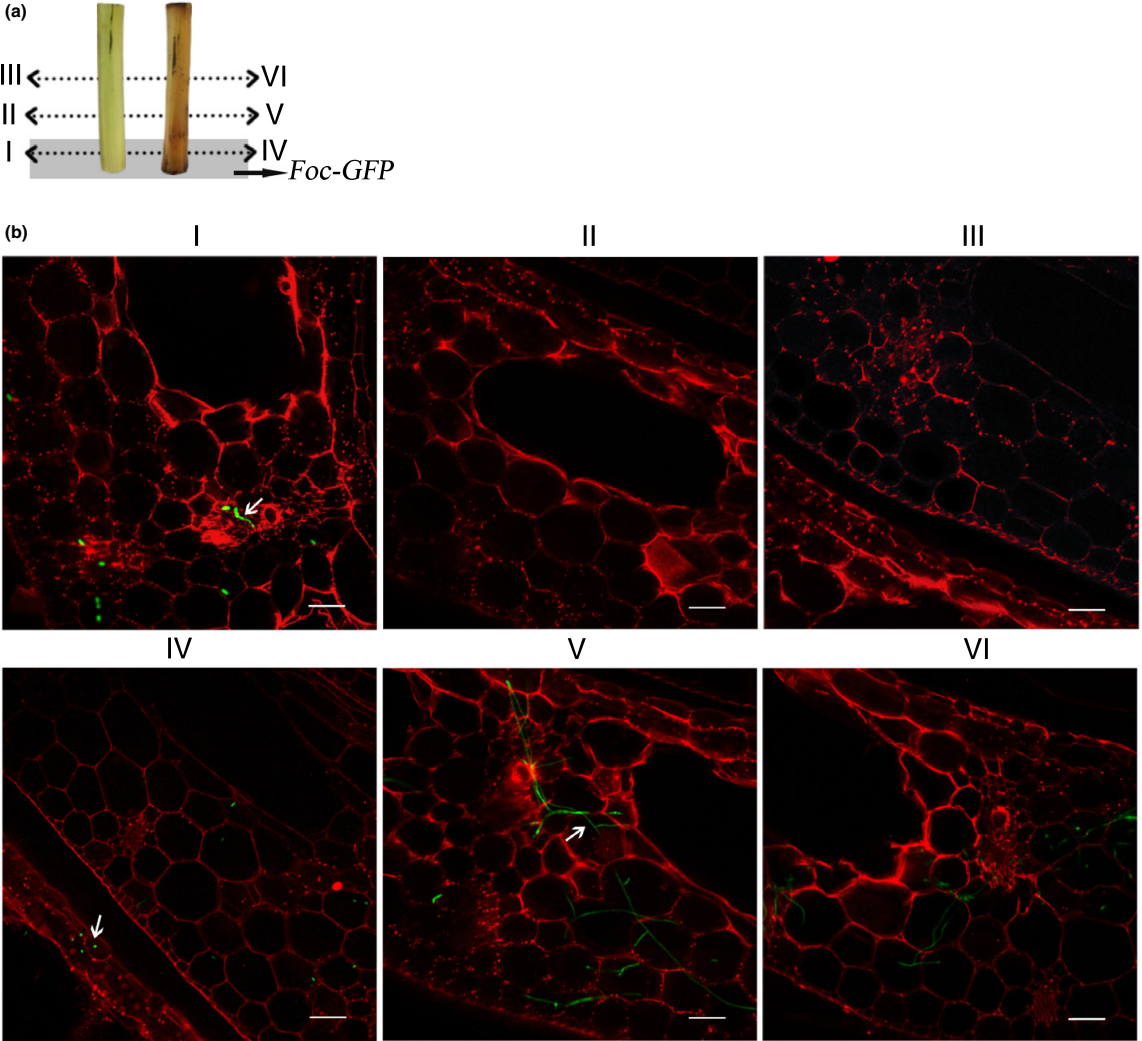
Cell death induced by fusaric acid (FSA) could accelerate *Fusarium oxysporum* f. sp. *cubense* tropical race 4 (*Foc* TR4) invasion. (a) Scheme of the analyzed zone; I–VI represent the corresponding observation sites of banana pseudostems, and grey shading symbolizes sections inoculated with *Foc*‐GFP (freen fluorescent protein). (b) Examination of infection process using GFP‐expressing *Foc* TR4 (green, arrows) at 12 h post‐inoculation. Bars, 100 μm.

In order to test what roles FSA plays during *Foc* TR4 invasion, GFP‐TR4 was used for the infection of banana pseudostems. Banana pseudostems treated with 300 μM FSA become corroded and water‐soaked, with clear necrosis symptoms, at 24 h post‐treatment, whereas the solvent‐treated pseudostems (control) developed only a natural browning over time (Fig. 5a). GFP‐TR4 was inoculated at the bottom of two different banana pseudostems to determine whether FSA‐induced histochemical necrosis can accelerate the spread of the pathogen into vascular tissues. At 12 h post‐inoculation (hpi) of GFP‐TR4, the GFP signal was detected at the middle of the FSA‐treated pseudostem, but it was only detected in the bottom of the solvent‐treated control pseudostem (Fig. 5b). This observation indicates that *Foc* TR4 can spread much faster when the tissue is treated with FSA.

### FSA regulates the expression of genes involved in mitochondria functions

In order to investigate the global gene expression affected by FSA, banana ECSs were treated with 300 μM FSA for 0, 6 and 24 h, and harvested for transcriptome analysis with RNA‐Seq. All assembled RNA‐seq sequences are publicly accessible through GenBank (accession no. SAMN11155476). Based on global RPKM‐expressing values, principal component and hierarchical cluster analysis showed that the three biological replicates of each sample were clustered into one group (Fig. S3a,b). In total, 14 395 genes were expressed; of these genes, 2581 were upregulated and 4736 were downregulated in the two treatment groups compared to the uninfected controls (Fig. S3c). Gene ontology (GO) terms associated with mitochondrion‐related genes, such as mitochondrial ribosome, mitochondrial inner membrane presequence translocase complex and mitochondrial respiratory chain complex IV, were enriched in the FSA‐treated banana ECSs (Figs S4, S5). In addition, cell death‐related genes were significantly enriched in the FSA‐treated samples. To confirm the RNA‐Seq data, qRT‐PCR was performed to analyze the expression of 10 genes individually, revealing an overall correlation between the two datasets (Fig. S6a,b). Consistent with the RNA transcript levels, the protein concentrations of HSP70 and AtpB in the 6 and 24 h FSA‐treated ECSs were reduced compared to those in the control (Fig. S6c). The nontreated ECS over 24 h accumulated to similar levels, showing that the increased accumulation of HSP70 and AtpB in FSA‐treated samples is not the reason for time extension. These results indicate that FSA may have significant impacts on mitochondrial function and cell death, and that a strong correlation exists between dysfunction of the mitochondria following FSA treatment and the downregulation of genes related to mitochondrial function.

### FSA inhibits mitochondria functions

**Figure 6:**
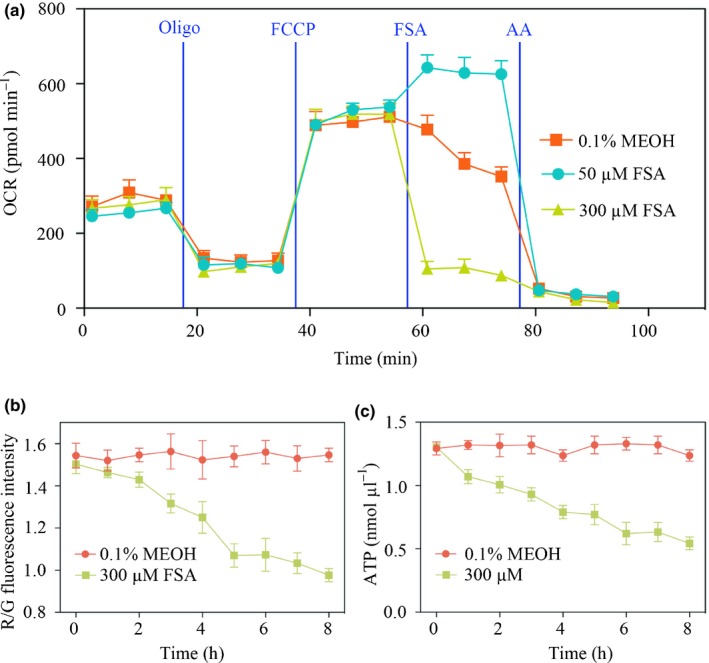
Effects of fusaric acid (FSA) on mitochondrial function of banana embryogenic cell suspensions protoplasts (ECSs). (a) Respiration measurements (OCR) in the presence of 8 μM oligomycin (Oligo), 6 μM FCCP and 5 μM antimycin A (AA), with 50 μM or 300 μM FSA and 0.1% MEOH control. (b) Banana protoplasts treated with 300 μM FSA showed decreased mitochondrial membrane potential (Δ*Ψ*m). Δ*Ψ*m was quantified by comparing the fluorescence intensity of red and green. (c) Banana protoplasts treated with 300 μM FSA showed decreased ATP contents. Data presented in (a–c) are means ± SD from three independent experiment.

Mitochondria produce the energy currency ATP for cells through respiration and to regulate cellular metabolisms. To investigate the effects of FSA on mitochondrial respiration, a mitochondrial stress test was performed to determine the OCR in banana ECS protoplasts in microtiter plates when FSA and other metabolism‐altering chemicals were added. Two different concentrations of FSA (50 or 300 μM) were used in two independent assays. Oligomycin (8 μM), FCCP (6 μM) and antimycin A (5 μM) were injected sequentially, and the OCR was measured after each injection. As expected, before the addition of the metabolism‐altering compounds, the protoplasts were in a quiescent state until the injection of oligomycin, which decreased the OCR due to the inhibition of ATP synthase (Fig. 6a). Next, the mitochondrial uncoupler FCCP stimulated the OCR by uncoupling the oxygen consumption from the ATP production (Fig. 6a). The lower concentration of FSA (50 μM) was found to further increase the OCR by acting as an uncoupler (Fig. 6a), whereas the higher concentration of FSA (300 μM) inhibited respiration to a relatively low OCR (Fig. 6a). Finally, antimycin A was injected to thoroughly shut down mitochondrial respiration (Fig. 6a). These results indicate that FSA could inhibit respiration, electron transport and ATP production in mitochondria.

The mitochondrion is a double membrane‐bound organelle, and a membrane potential across the inner membrane is formed by the actions of electron transport chain enzymes. Therefore, the loss of the mitochondrial membrane potential is an early indication of mitochondria dysfunction. The dye JC‐1 was used as a marker to monitor the mitochondrial membrane potential in banana protoplasts, testing the effect of FSA, because JC‐1 changes its color from red to green when membrane potential decreases. It was found that the mitochondrial membrane potential (Δ*Ψ*m) was significantly decreased after the FSA treatment by c. 61.3% over 8 h (Fig. 6b). The change in the ATP content in the FSA‐treated banana protoplasts was also recorded. Likewise, compared to the 0.1% MEOH‐treated controls, the ATP content in FSA treated banana protoplasts decreased by *c*. 58.7% over 8 h (Fig. 6c).

### FSA induces ROS production

**Figure 7:**
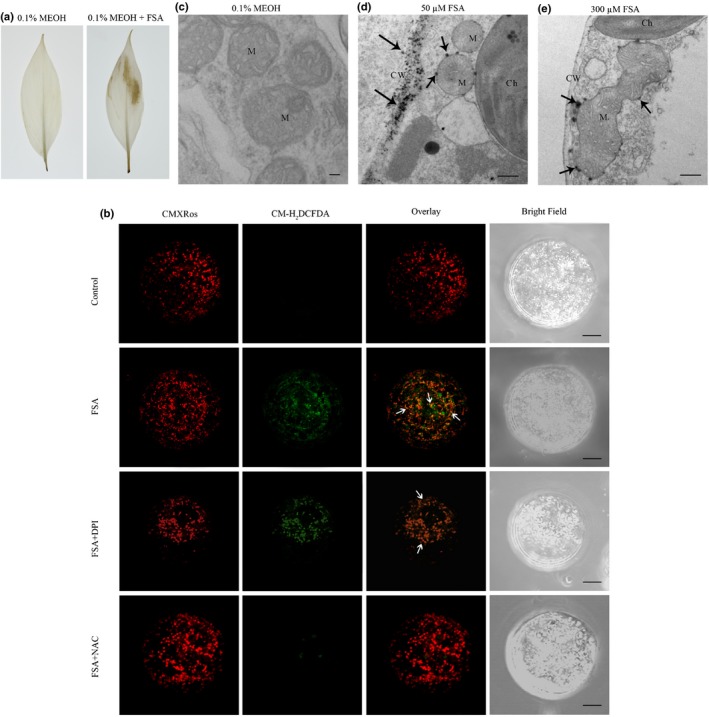
Reactive oxygen species (ROS) detected in fusaric acid (FSA)‐treated banana leaves and embryogenic cell suspension (ECS) protoplasts. (a) Leaves were treated with 0.1% MEOH or 300 μM FSA and stained with DAB as described in the Materials and Methods section. The brown precipitate indicated DAB polymerization at the site of ROS production. (b) Confocal laser scanning microscopy of protoplasts from 7‐d‐old banana ECSs treated with 0.1% MEOH (control), FSA (300 μM), FSA + DPI (inhibitors of NADPH oxidase), and FSA + NAC (a ROS scavenger) for 5 min. Samples were double‐stained with CM‐H_2_ DCFDA (to detect ROS) and CMXRos (mitochondrial marker). (c) to (e) Electron microscopy localization of H_2_O_2_ production in banana tissue culture seedlings treated with 0.1% MEOH (c), 30 μM FSA (d) or 50 μM FSA (e). Three or more different leaf samples of both 0.1% MEOH‐treated and FSA‐treated plants were used in each experiment. Images represent typical observations in two independent experiments. Arrows indicate depositions of cerium. Bars: (b) 10 μm; (c) 200 nm; (d, e), 1 μm. M, mitochondrion; CW, cell wall; Ch, chloroplast.

Necrotic lesions (Li *et al*., 2013b) are among the first symptoms after FSA treatment resembling the hypersensitive response (HR). To examine whether the FSA treatment promotes the production of ROS, an important component of the HR (Lamb & Dixon, 1997), banana leaves were treated with FSA and examined within an hour for ROS production using DAB staining. The treated leaves generated a brown precipitate indicative of ROS production (Fig. 7a), and this precipitate was completely absent in the mock‐treated leaves.

The treatment of protoplasts isolated from 7‐d‐old banana ECS with 300 μM FSA produced ROS within 5 min (Fig. 7b). Interestingly, as detected by CM‐H_2_DCFDA staining, the ROS colocalized with mitochondria labeled by CMXRos (Fig. 7b). Mitochondria and the cytoplasm are the two major sources of ROS in cells (Shen, 2010). ROS in the cytoplasm is catalyzed by NADPH oxidase (NOX), which can be inhibited by DPI. To explore which source contributes to the generation of FSA‐induced ROS, protoplasts were first co‐incubated with FSA and DPI. Interestingly, ROS was still detectable and colocalized with the mitochondria, although to a lesser extent (Fig. 7b), indicating that the cytosolic inhibition of ROS production was not able to block the FSA‐induced ROS production. The coincubation of cells with FSA and NAC, an H_2_O_2_ scavenger, however, resulted in barely detectable ROS signals (Fig. 7b). Therefore, in banana protoplasts treated with FSA, mitochondria seem to be the primary sites of ROS production.

Inhibition of the ATP synthase would increase the ROS production subsequently (Tiwari *et al*., 2002). To detect the sites of H_2_O_2_ production induced by FSA in the mitochondria, histochemical staining was performed using cerium chloride (CeCl_3_), which reacts with H_2_O_2_ to produce electron‐dense precipitates of cerium perhydroxide that can be visualized using electron microscopy (Yao *et al*., 2002). As expected, H_2_O_2_ was not detected in the mock inoculations (Fig. 7c), but in the banana tissue culture plantlets treated with 50 μM FSA, the widespread accumulation of H_2_O_2_ was more evident in the plasma membrane, cell wall and intercellular spaces of the leaf cells. A relatively low number of spots scattered along the mitochondrial outer membrane (Fig. 7d). In addition, several dark spots were detected within mitochondria but were not sufficient to cause mitochondrial or cellular destruction (Fig. 7d). However, following treatment with 300 μM FSA, extensive patches of CeCl_3_ precipitates were detected on the inner and outer membranes of mitochondria (Fig. 7e). At this time, H_2_O_2_ accumulation was evident in the mitochondria and was accompanied by a deterioration in the outer and inner mitochondrial membranes, resulting in a change in the general structural integrity of the mitochondria (Fig. 7e).

### FSA promotes lesion formation through ROS production

**Figure 8:**
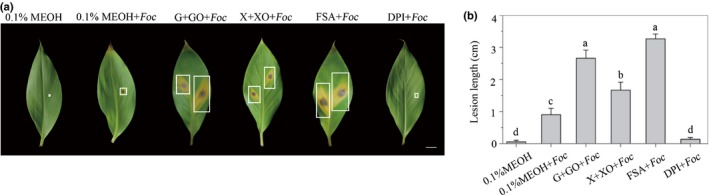
Fusaric acid (FSA) promoted lesion formation of *Fusarium oxysporum* f. sp. *cubense* tropical race 4 (*Foc* TR4) in banana leaves through reactive oxygen species (ROS) production. (a) Banana leaves were infiltrated with 0.1% MEOH or *Foc* TR4 together with 0.1% MEOH, mixture of glucose oxidase (25 units ml^−1^) plus 2.5 mM glucose (G+GO), mixture of xanthine oxidase (0.05 units ml^−1^) plus 0.5 mM xanthine (X+XO), 300 μM FSA, or 10 μM DPI. *Foc* TR4 was inoculated in the center of the infiltrated area 2 h later. Photographs were taken 5 d post‐inoculation (dpi). The white squares indicate cell death lesions in leaves. Bar, 1 cm. (b) Disease progress was evaluated at 5 dpi by measuring lesion length. The data are shown as the means ± SD from three independent experiments and different letters above the columns indicate the significant difference (*P* < 0.05) between different treatments.

Production of ROS is a plant defense response that leads to necrosis formation. To examine whether FSA enhances the *Foc* TR4 infection by inducing a ROS burst to promote the lesion formation in the plants, the concentrations of ROS in leaves were experimentally altered. The infiltration of G+GO induces the host to produce H_2_O_2_, and X+XO induces the host to produce O^2–^ (Govrin & Levine, 2000). It was found that the infiltration of both mixtures greatly enhanced the lesion formation in banana leaves when co‐inoculated with *Foc* (Fig. 8a). Consistent with previous reports (Taheri *et al*., 2014), no lesion was detected in the leaves infiltrated with ROS‐producing mixtures alone without the pathogen (data not shown). Necrosis also was strongly induced by the presence of FSA together with *Foc* (Fig. 8a). The infiltration of 3 mM DPI, an inhibitor of the NADPH oxidase, into the intercellular spaces before *Foc* TR4 inoculation diminished the ROS‐induced necrosis to a point where it was indistinguishable from the control (Fig. 8a), suggesting that the enhancement of disease symptom development by FSA may be mediated by the generation of ROS. Compared to DPI treatment and the control, treatment of the banana leaves with FSA or G+GO before inoculation with *Foc* TR4 resulted in significantly longer lesion lengths (Fig. 8b).

### FSA induces apopotosis phenotypes

**Figure 9:**
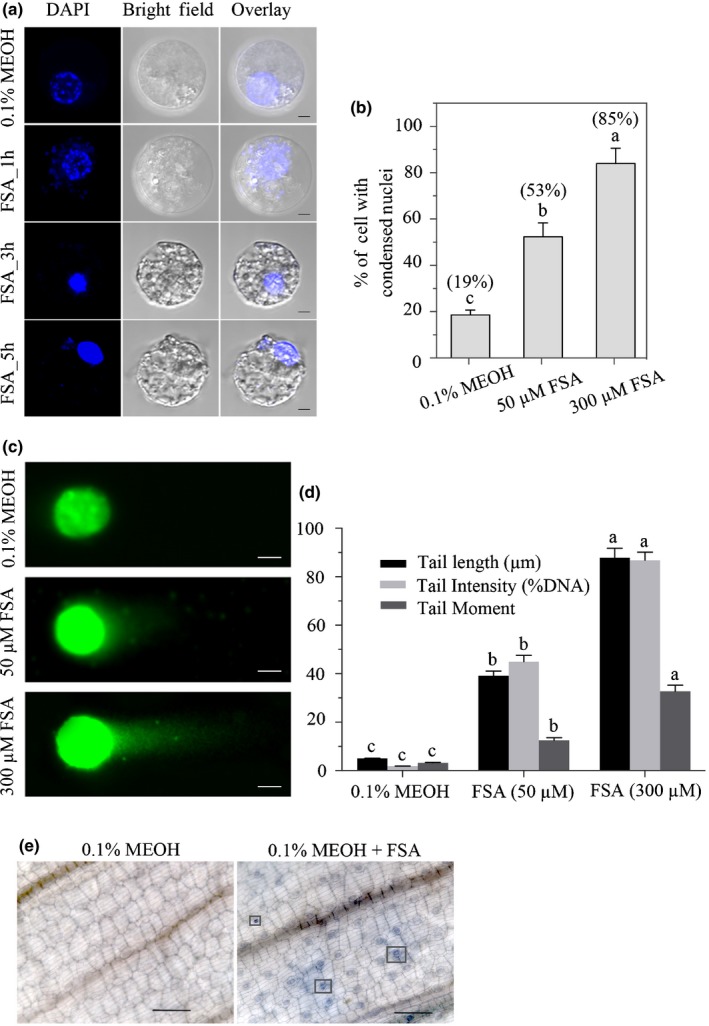
Fusaric acid (FSA) induced apoptosis symptoms in banana embryogenic cell suspension (ECS) protoplasts and leaf cells. (a) Protoplasts isolated from 7‐d‐old banana ECS were treated with 0.1% MEOH or 300 μM FSA for 24 h and stained with DAPI (nuclear marker). Images were taken by confocal laser scanning microscopy. At least 300 protoplasts were observed. This experiment was repeated three times with similar results. Bars, 10 μm. (b) Effect of FSA on the induction of nuclear condensation in banana protoplasts after 24 h of incubation. Percentages in brackets refer to the increase in protoplasts with condensed nuclei in relation to the control (0.1% MEOH). (c, d) DNA comet assay. (c) The body of banana protoplasts treated with 0.1% MEOH, 50 μM FSA and 300 μM FSA. Bars, 10 μm. (d) Changes of DNA tail length (μm), tail intensity (%) and tail moment when protoplasts treated with FSA. Data presented in (b, d) are the means ± SD from three independent experiments and different letters above the columns indicate the significant difference (*P* < 0.05) among different treatments at the same point. (e) Microscopic visualization of cell death on leaves treated with 0.1% MEOH, 300 μM FSA for 1 h using Trypan blue staining. Boxes indicated the death of the stomata guard cells. Bars, 100 μm.

In order to identify additional phenotypes, a nuclear counter‐stain DAPI (4′‐6‐diamidino‐2‐phenylinodle) was used to observe the impact of FSA on potential nuclear condensation. Although the control cells treated with 0.1% MEOH showed normal nuclei (smooth nuclear), the FSA‐treated banana protoplasts showed apoptotic nuclei with condensed chromatins (Fig. 9a). Compared to the control, at 24 hpi, both concentrations of FSA (50 and 300 μM) significantly increased the nuclear condensation in the protoplasts, by 35% and 67%, respectively (Fig. 9b). The bodies of the normal banana protoplasts showed intact, undamaged DNA that remained in the region of the nuclear matrix. However, the banana protoplasts exposed to 50 or 300 μM FSA suffered damage and assumed a comet‐like appearance, including a ‘tail’ shape (Fig. 9c,d). In addition, significant differences in the comet‐like characteristics caused by the two different FSA dosages suggest that FSA could directly lead to DNA strand breakage. Therefore, it is concluded that the FSA treatment of banana protoplasts led to apoptosis phenotypes of nuclei fragmentation and chromatin condensation. To better visualize the occurrence of cell death resulted from apoptosis, the leaves were stained with trypan blue (Fig. 9e). By 1 d after FSA infiltration, treated leaves displayed cell death around the infiltration site and in stomata guard cells, whereas the mock‐treated leaves were symptomless.

## Discussion

Fusaric acid (FSA) is produced at high concentrations in many phytopathogenic and nonpathogenic *Fusarium* strains (Kachlicki & Jedryczka, 1997). The identification of the Fusaric acid biosynthetic (*FUB*) cluster and the creation of their deletion and complementation strains have facilitated our understanding of the impact of FSA on phytotoxicity. Brown and colleagues deleted nine of the 12 genes to inhibit FSA production(Brown *et al*., 2015). However, their results showed that the lack of FSA production did not affect the virulence of *F. oxysporum* on cactus or *F. verticillioides* on maize seedlings (Brown *et al*., 2015). In the present study, the inactivation of *FUB1*,* FUB3*,* FUB4* and *FUB10* in *Fusarium oxysporum* f. sp. *cubense* (*Foc*) ‘tropical’ race 4 (TR4) reduced the production of FSA both *in vitro* and *in vivo*, when tested on banana plantlets (Fig. 2). In addition, the results reported herein showed that FSA is important for the virulence of *Foc* TR4.

In previous pathogenicity tests, it was found that a higher concentration of spores in the soil reduced the ability of *Foc* TR4 to discriminate among the resistant, tolerant and sensitive banana varieties or germplasm (data not shown). At high concentrations, it was difficult to find a difference in pathogenicity between the *fub* mutants and the wild‐type (WT) because they were both able to induce disease rapidly in the banana plantlets. However, by decreasing the inoculation concentration of the *fub* mutants in the soil, delayed wilting symptoms and a decreased disease index were noticed (Fig. 3). Therefore, a high concentration of spores and mycelium used in the inoculation must have changed the host–pathogen interactions and masked the function of FSA in the infection of *Foc* TR4.

Both previous results (Brown *et al*., 2015; Studt *et al*., 2016) and the inoculation results reported herein showed that the *fub* mutants did not lose their virulence completely, and could still infect and develop disease symptoms in the host plants (Fig. 3). These observations indicate that FSA is not the only virulence factor of *Fusarium* and that other factors may act in a similar way to FSA and substitute for its roles in the pathogenicity. Based on the published *Foc* TR4 II5 genome sequence, it was predicted that *c*. 500 effectors are secreted into host tissues (data not shown). Similar to FSA and SECRETED INTO XYLEM (SIX)1 (Rep *et al*., 2005) that cause necrosis in host tissues, SIX is secreted into the xylem (Houterman *et al*., 2007) and travels upward in sap to aid the infection process. In addition to FSA, *F. oxysperum* also may produce beauvericin, bikaverin, enniatins, fusarin C, isoverrucarol, moniliformin, naphthoquinone pigments, sambutoxin and wortmannin (Jennings, 2007). Although the effects of these toxins are not tested in banana cells and their roles during infection are not yet characterized, the possibility cannot be excluded that they may have overlapping roles with FSA in pathogen virulence. When the FSA production is inhibited, these other toxins may be able to compensate the defects.

In the present study, it was found that FSA is a pioneer molecule during pathogenesis, diffusing to host tissue before the arrival of the invading *Foc* TR4 hyphae. At 12 d post‐inoculation (dpi), no fungal colonization was observed in the banana pseudostems, but FSA was already present in the pseudostems as early as 6 dpi (Fig. 4). Using quantitative (q)PCR combined with liquid chromatography‐tandem mass spectrometry (LC‐MS/MS), it was confirmed that FSA accumulated, but without detecting any *Foc* TR4, in the upper parts of plants when only the roots were inoculated. However, the mechanism behind this observation was unclear. Deoxynivalenol (DON) is the predominant phytotoxin in cereal grains infected with *Fusarium* species (Placinta *et al*., 1999). When labeled with fluorescein as a tracer, the vascular transportation of DON was demonstrated by histological studies (Winter *et al*., 2013). Because FSA is water soluble, when it is produced in the root systems in significant amounts, it perhaps can be transported upward passively in the aqueous phase via the transpiration stream in xylem vessels over a long distance.

The FSA secreted by *F. oxysporum* f. sp. *radicis‐lycopersici* is a good chemo‐attractant of its biocontrol strain *Pseudomonas fluorescens* WCS365 in spent growth media(de Weert *et al*., 2004). Therefore, one can assume that FSA might have served as a chemo‐attractant that guides the upward infection of *Foc* TR4. However, when tested in a plate‐based assay (data not shown), neither a chemotactic response in *Foc* TR4 nor growth of the fungus toward FSA were found. Because of these observations, the present authors believe that FSA produced in *Foc* TR4 is not an allelochemical or chemotaxin. Instead, it is more likely a virulent molecule adventuring into the uninfected tissues first and preparing for the upward progression of the pathogen invasion.

Although there were several studies investigating the mode of action of FSA (Bouizgarne *et al*., 2006b; Samadi & Shahsavan Behboodi, 2006; Jiao *et al*., 2013), the cellular targets of FSA and its precise function in fungal pathogenesis remain unclear. One of the first responses to pathogen attack is the generation of an oxidative burst that can trigger cell death as a hypersensitive response (HR) (Lamb & Dixon, 1997), which facilitates the rapid growth and spread of necrotrophic plant pathogens (Govrin & Levine, 2000). Taking advantage of the RNA‐Seq transcriptome analysis, it was found that > 5000 genes were regulated in the FSA‐treated banana embryogenic cell suspensions (ECSs) and that the differentially expressed genes primarily were enriched in the oxidative phosphorylation and apoptosis signaling pathways.

Several studies have provided evidence that FSA can cause mitochondrial dysfunction and cell death in plant and mammalian hosts (Telles‐Pupulin *et al*., 1998; Bouizgarne *et al*., 2006a; Samadi & Shahsavan Behboodi, 2006; Jiao *et al*., 2013). It is shown that higher concentrations of FSA (> 10^−4^M) are toxic to plants and can inhibit both O_2_ uptake and host cell growth by increasing the membrane permeability and inhibiting mitochondrial activity (Bouizgarne *et al*., 2006a). Mitochondrial respiration was measured to reveal the transient changes induced by FSA and other compounds. The results showed that FSA not only inhibited O_2_ uptake, but also acted as a respiratory uncoupler in a dose‐dependent manner (Fig. 6). When FSA was applied to banana ECS protoplasts at low concentration (< 10^−5^M), O_2_ uptake was stimulated (Fig. 6), with moderate accumulation of reactive oxygen species (ROS) and lower cell death rate compared to treatment at a high concentration (data not shown). This result may be explained by a previous study which showed that FSA could act as an elicitor of early defense and induce phytoalexin synthesis (Bouizgarne *et al*., 2006b). The high concentration of FSA caused mitochondrial dysfunction, accompanied by high accumulation of ROS (Fig. 7), which mimics the specific inhibition of mitochondrial electron transport by antimycin A.

Many studies have implied ROS as the direct or indirect mediator of the programmed cell death (PCD). Mitochondria play key roles in apoptosis, especially by mediating ROS eruption (Yao *et al*., 2002; Win *et al*., 2012). In the present study, it was shown that the function of mitochondria was dramatically altered by the FSA treatment, which was accompanied by nuclear condensation (Fig. 9a). It also was demonstrated that PCD plays a role in an activation mechanism when banana leaves are exposed to FSA, and significant ROS accumulation and cell death were detected (Figs 7a, 9e). Therefore, it seems that FSA is dispersed throughout plant tissues to disturb mitochondrial functions and induce ROS overproduction, which ultimately leads to host cell death and facilitates the necrotrophic infection of the host plants.

**Figure 10:**
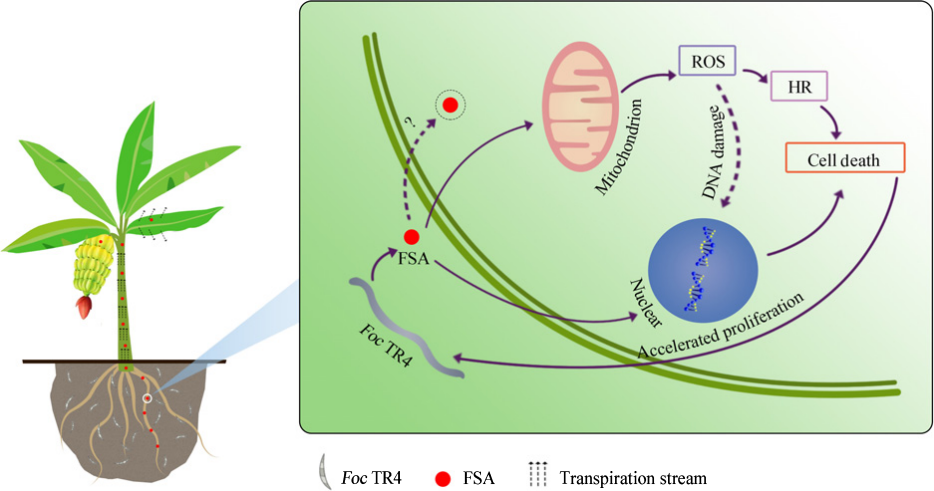
A model depicting the role of fusaric acid (FSA) produced by *Fusarium oxysporum* f. sp. *cubense* tropical race 4 (*Foc* TR4) in disease development. FSA secreted by *Foc* TR4 disturbs mitochondria functions by inducing the production of mitochondrial reactive oxygen species (ROS), which in turn triggers a hypersensitive reaction (HR). As a result, host cells show FSA‐induced cell death. These changes accelerate the growth of *Foc* TR4 from the root into the xylem with accelerated proliferation rate. FSA also might transport upward in the aqueous phase via the transpiration stream in the xylem vessels over a long distance, even reach the banana fruits.

In summary, *Foc* TR4 may employ an array of strategies to accelerate infection. These strategies include the secretion of a pioneer molecule FSA, which can diffuse ahead of the pathogen and compromise the host's immunity by disturbing host respiration and inducing mitochondrial ROS production, toxin‐induced HR or cell death (Fig. 10). These changes facilitate the invasion of *F. oxysporum* from the root into the xylem and aid the development of *F. oxysporum*‐induced symptoms such as vascular wilt, damping‐off and root rot in banana.

## Author contributions

CL, GY and LM conceived the study and designed the experiments; SL, JL, YZ and NL performed the experiments; CL and GY were involved in funding acquisition; AV, DM, CZ, CH, FB, HG and OS analyzed the data; GD, QY, Tao Dong and Tongxin Dou were involved in obtaining the materials; CL and SL wrote the manuscript with contributions from the other authors; and SL, JL and YZ contributed equally to this work.

## Supporting information

Please note: Wiley Blackwell are not responsible for the content or functionality of any Supporting Information supplied by the authors. Any queries (other than missing material) should be directed to the *New Phytologist* Central Office.

**Fig. S1** Generation of *fub* mutants.
**Fig. S2** Relative expression of *FUB* genes.
**Fig. S3** Global transcriptome analysis based on RNA‐seq.
**Fig. S4** Enriched pathways among all genes that are significantly up‐/downregulated by FSA treatments.
**Fig. S5** Enriched genes associated with mitochondrial function and cell death in 6 h or 24 h FSA‐treated samples.
**Fig. S6** Verification of RNA‐seq data by qRT‐PCR and Western blotting.
**Table S1** The primer used in this study.
**Table S2** Abbreviation list in this study.
