# Supplementary material for: Fusaric acid instigates the invasion of banana by Fusarium oxysporum f. sp. cubense TR4
Source: New Phytol. 2019 Oct 24;225(2):913–29. doi: 10.1111/nph.16193 (PMC6973005; doi:10.1111/nph.16193)
Supplement: Supplementary file 1 — Fig. S1 Generation of fub mutants. Fig. S2 Relative expression of FUB genes. Fig. S3 Global transcriptome analysis based on RNA‐seq. Fig. S4 Enriched pathways among all genes that are significantly up‐/downregulated by FSA treatments. Fig. S5 Enriched genes associated with mitochondrial function and cell death in 6 h or 24 h FSA‐treated samples. Fig. S6 Verification of RNA‐seq data by qRT‐PCR and Western blotting. Table S1 The primer used in this study. Table S2 Abbreviation list in this study. [file NPH-225-913-s001.pdf]

New Phytologist **Supporting Information**

Article title: Fusaric acid instigates the invasion of banana by *Fusarium oxysporum* f. sp. *cubense* TR4
Authors: Siwen Liu[2,*], Jian Li[4,*] , Yong Zhang[5,*], Na Liu[3], Altus Viljoen[6]， Diane Mostert[6], Cunwu Zuo[1], Chunhua Hu[1], Fangcheng Bi[1], Huijun Gao[1], Ou Sheng[1], Guiming Deng[1], Qiaosong Yang[1], Tao Dong[1], Tongxin Dou[1], Ganjun Yi[1,†], Li-Jun Ma[5,†] & Chunyu Li[1,†]
Article acceptance date: September, 5th, 2019

The following Supporting Information is available for this article:

Fig. S1 Generation of *fub* mutants (a) Schematic representation of the disruption strategy for *FUB1*, *FUB2*, *FUB3*, *FUB4*, *FUB5*, and *FUB10.* Positions of the primers are marked as arrows. **(b)** Southern blotting analysis of *fub* mutants with a fragment of the hygromycin resistance gene as the probe. *Pst*I was used for genomic DNA digestions and represented by vertical bars as the recognition sites. The expected sizes of the resulting hybridization bands are indicated. **(c)** Colony of the wild type (II5), the *fub* mutants and the complemented (*fub*-C) strains grown on PDA at 28°C for 4 d.

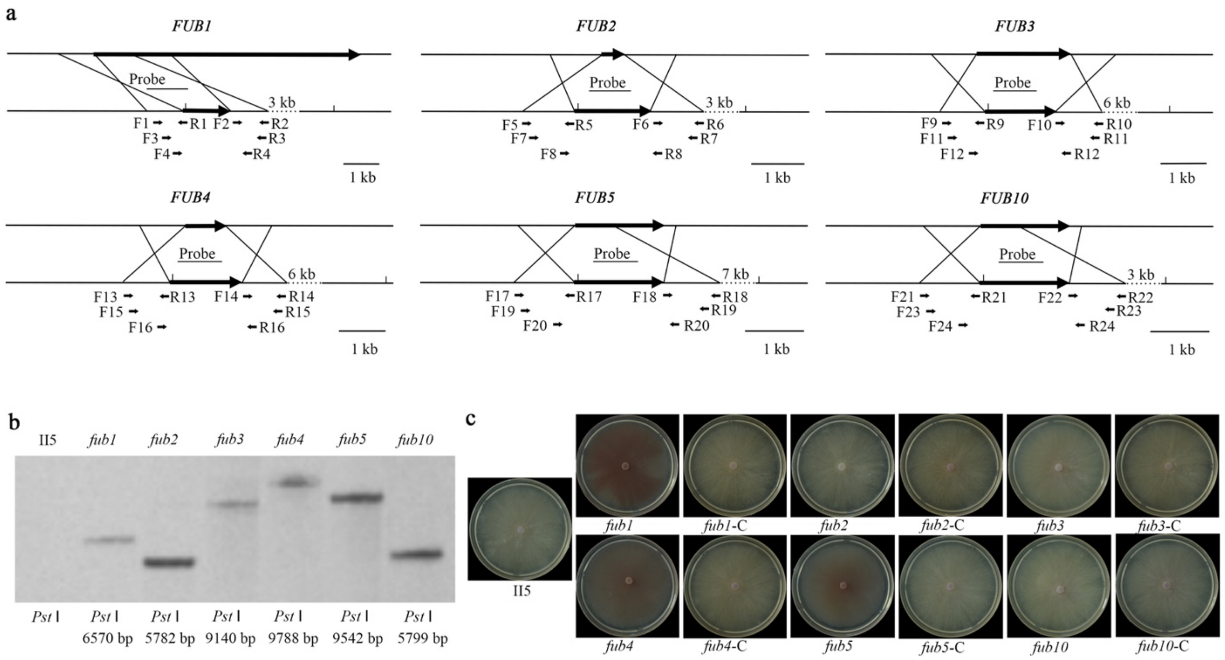

Fig. S2 Relative expression of *FUB* genes. WT and *fub* mutant strains were grown in fusaric acid producing broth (Czapek-Dox broth [CDB]) for 9 days. Values represent the means ± SD from three independent experiments. Different letters above the columns indicate the significant difference ($p < 0.01$) between WT and mutant strains at the same time point.

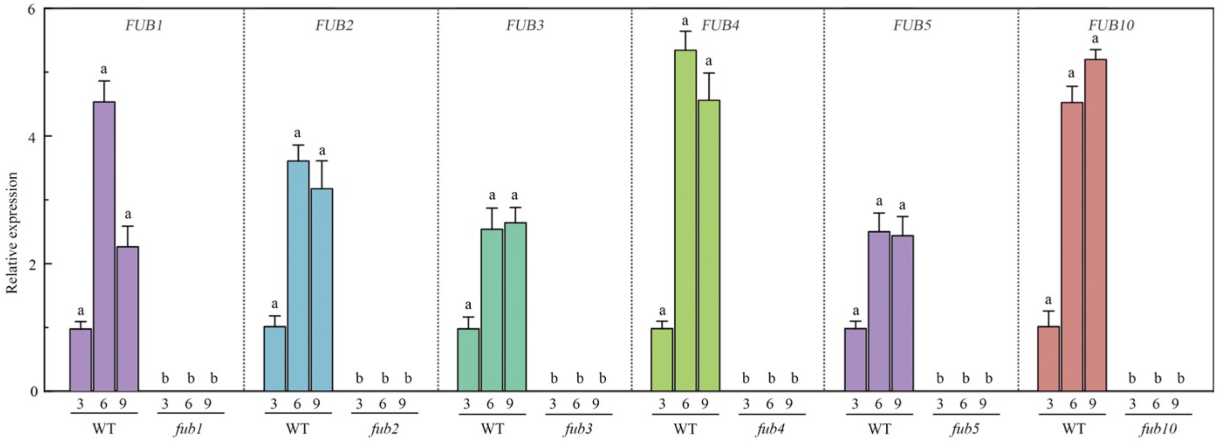

Fig. S3 Global transcriptome analysis based on RNA-seq. The Cavendish banana embryogenic cell suspensions (ECSs) were treated with FSA for 6 h or 24 h. **(a)** Principal component analysis. **(b)** Hierarchical cluster analysis. The color scale represents the Pearson correlation. **(c)** The number of genes differentially expressed in 6 h FSA-treated and 24 h FSA-treated Cavendish banana ECSs.

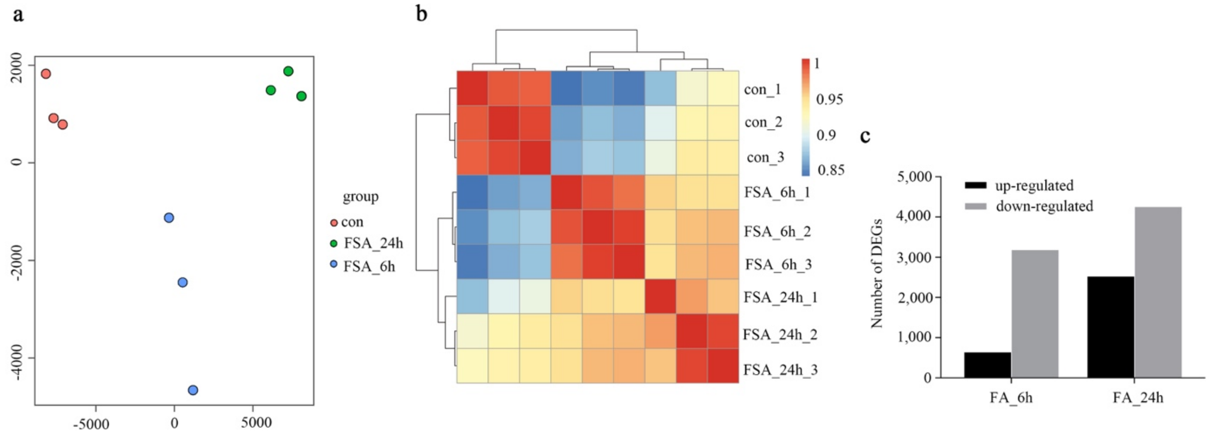

Fig. S4 Enriched pathways among all genes that are significantly up/down regulated by FSA treatments. (a) 6 h FSA-treated sample. **(b)** 24 h FSA-treated sample.

a

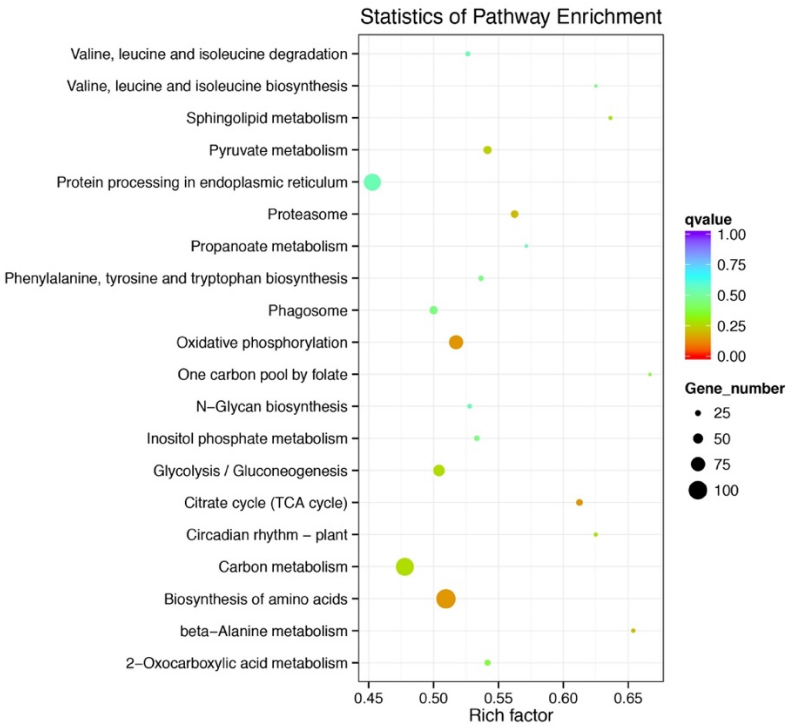

b

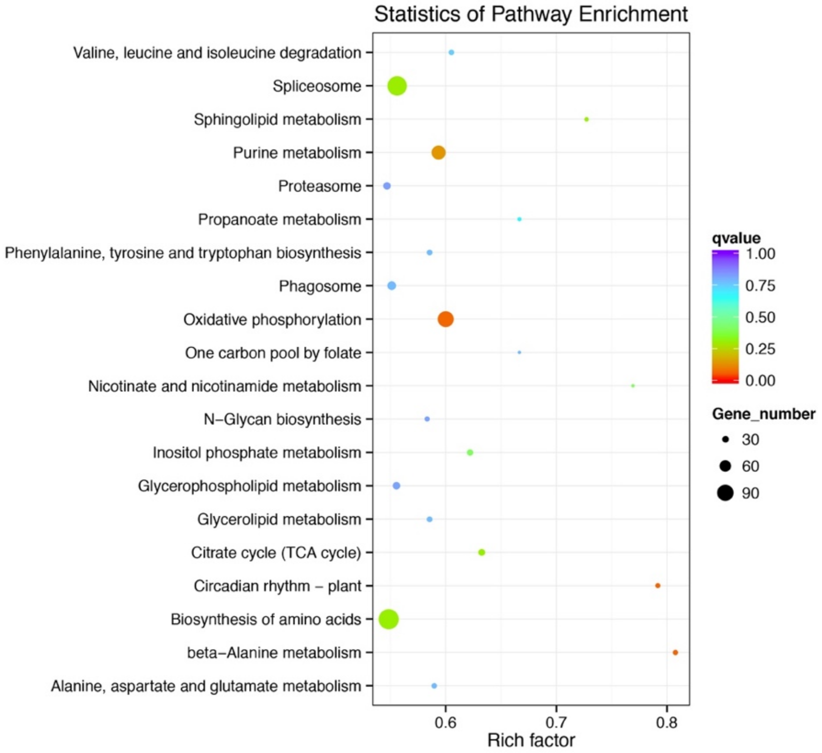

Fig. S5 Enriched genes associated with mitochondrial function and cell death in 6 h (a) or 24 h (b) FSA-treated samples.

a

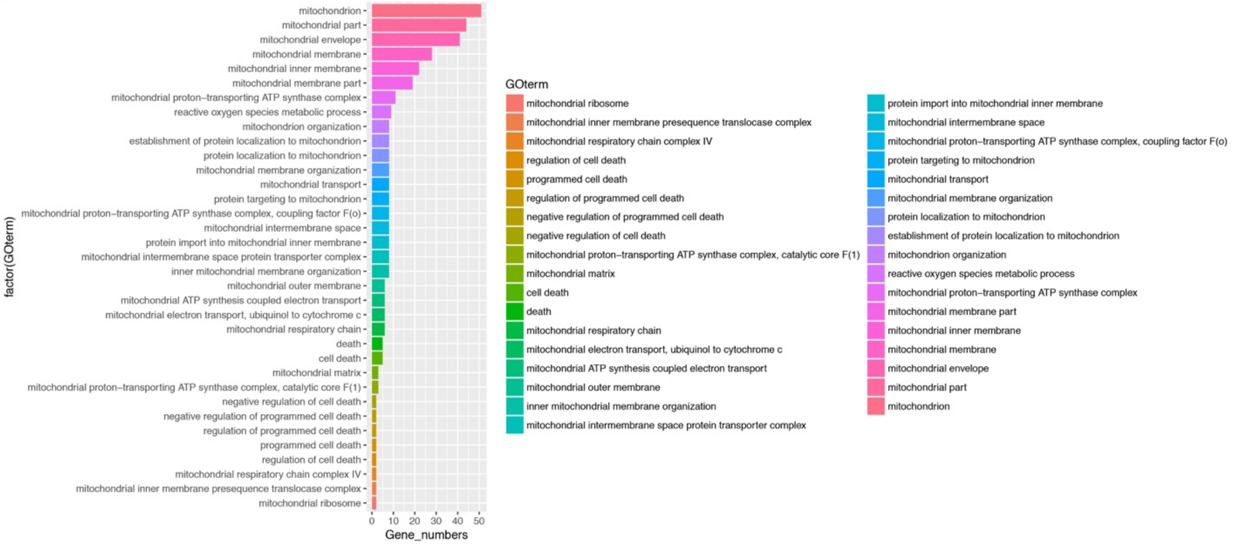

b

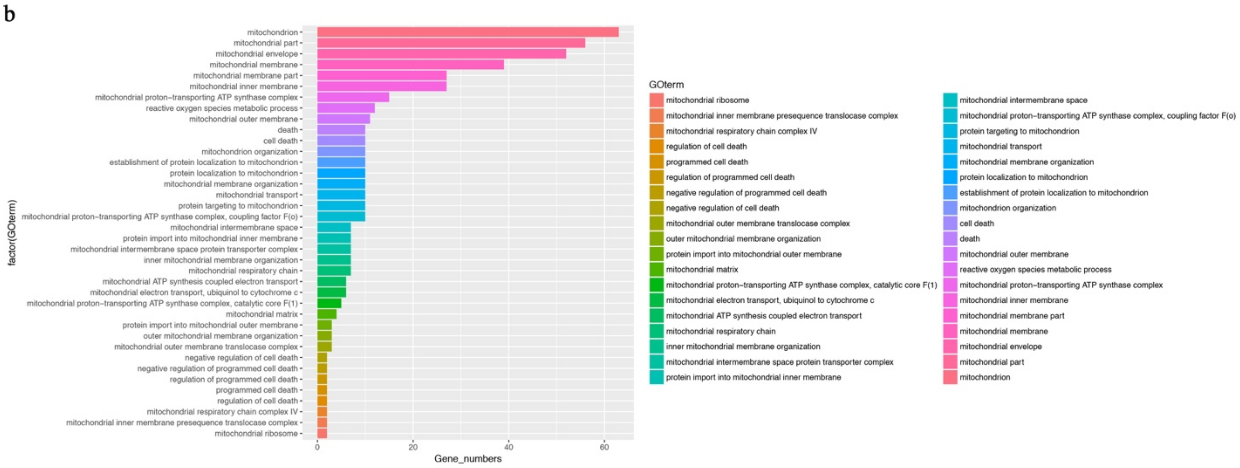

Fig. S6 Verification of RNA-seq data by qRT-PCR and western blotting. (a) and **(b)** Comparison of RNA-seq based gene expression and qRT-PCR verification. Values are means ± SD from triplicate biological repeats. **(c)** Western blotting analyses of Hsp70 and AtpB proteins in 6 h and 24 h FSA-treated Cavendish banana ECSs.

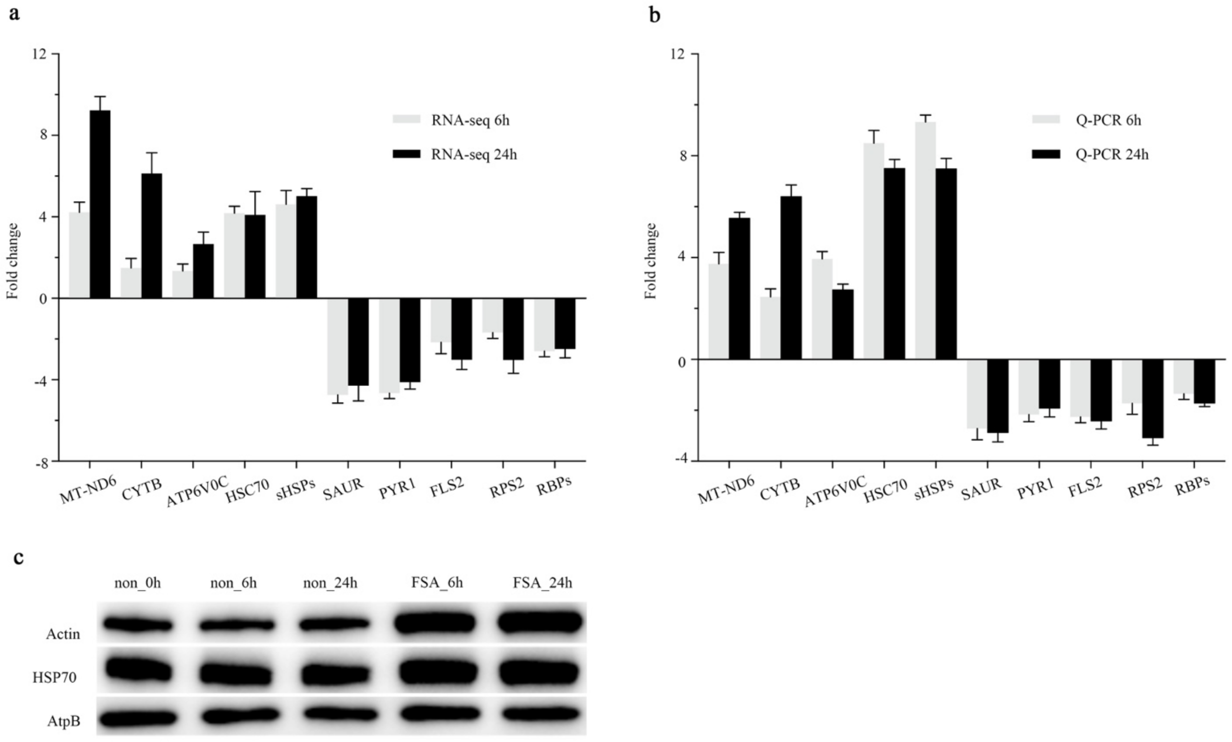

Table S1 PCR primers used in this study

Oligo name	Sequence
FOIG_16450-UP-F[a]	TGCCTATTTATTTATTAAACTCAGCCTTG
FOIG_16450-UP-R[a]	CAAAATAGGCATTGATGTGTTGACCTCCTGTGAGAGGAAAAAGAGTCAGAAAC
FOIG_16450-DOWN-F[b]	CTCGTCCGAGGGCAAAGGAATAGAGTAGCACGCATGCAGCCAG
FOIG_16450-DOWN-R[b]	CGTAGGGGGGAAGATCGACGA
FOIG_16450-NEST-F[c]	TATCTACTATAGACCAGATGGCTAGATCT
FOIG_16450-NEST-R[c]	GGTCCCTGAAGAGCAGCG
FOIG_16450-ID-F[d]	AACCCGGTCTAGCTAGCT
FOIG_16450-ID-R[d]	TTGGCATAAGCAGCAGCAATC
FOIG_16450-COM-F[e]	TTT CGT AGG AAC CCA ATC TTC AAA
FOIG_16450-COM-R[e]	CACCACCCCGGTGAACAGCTCCTCGCCCTTGCTCACATGACTCTCTCCAATGGCTC
FOIG_16450-Q-F[f]	CGACAACAGATGCCACTCCT

FOIG_16450-Q-R[f]	GCTGAAGCATAAGTCGCACG
FOIG_16451-UP-F	CTCGCGAAAGTCGCTGCGTCTTCCAGCAATCCCTT
FOIG_16451-UP-R	CAAAATAGGCATTGATGTGTTGACCTCCGATGGGCAGTGACAGTGACT
FOIG_16451-DOWN-F	CTCGTCCGAGGGCAAAGGAATAGAGTAGTTTAGTGGAGAGGAACGCG
FOIG_16451-DOWN-R	TTGATGATCGTGATTAGTAGTTTGAGC
FOIG_16451-NEST-F	TCGCGCAATGTGTATAGATTGGT
FOIG_16451-NEST-R	CTCGGCAACCTACCATATTCATG
FOIG_16451-ID-F	ACTAAGATAGTTCGGCCAGCAT
FOIG_16451-ID-R	GAACATGACGAGGCCGATCG
FOIG_16451-COM-F	CACCACCCCGGTGAACAGCTCCTCGCCCTTGCTCACATGGCCACCGAGCTCAAAG
FOIG_16451-COM-R	CTCGCGAAAGTCGCTGCGGCCTTTCAAGAGTAAATAA
FOIG_16451-Q-F	ACCTCGTCATCGTCCCTGAT
FOIG_16451-Q-R	TGTTCCATTCTCGGCGACTT
FOIG_16452-UP-F	ATCAACGATATCTCAGTCACTGTCACT
FOIG_16452-UP-R	CAAAATAGGCATTGATGTGTTGACCTCCCCTGGCAAGTGTTGTTACG
FOIG_16452-DOWN-F	CTCGTCCGAGGGCAAAGGAATAGAGTAGATATAGCTGTAAGGGATTAAGCAC
FOIG_16452-DOWN-R	GCCGACGTACCTTCCCG
FOIG_16452-NEST-F	GCCAACAAGTCGCCGAG
FOIG_16452-NEST-R	CTTCAGTTCATGTTCGTGGCT
FOIG_16452-ID-F	GGGGACTTCTCGCGATAAGA
FOIG_16452-ID-R	CACCGCCCATGGACTAACT
FOIG_16452-COM-F	CACCACCCCGGTGAACAGCTCCTCGCCCTTGCTCACATGCGCAATCGTCGAGATAA
FOIG_16452-COM-R	CTCGCGAAAGTCGCTGCTACGAAGAACACCATATAG
FOIG_16452-Q-F	CAGTCCATGTCTCACGGCTT
FOIG_16452-Q-R	TTCTTCGGCAAGTCTGCACT
FOIG_16453-UP-F	ACCGGATTCGAGTGTGAAGTT
FOIG_16453-UP-R	CAAAATAGGCATTGATGTGTTGACCTCCCTTGATCGAATCTAAATGGCTTTT
FOIG_16453-DOWN-F	CTCGTCCGAGGGCAAAGGAATAGAGTAGATACTCATTGTTGTTTTCTTAT
FOIG_16453-DOWN-R	CTATGGCAATCAACAGTTCCAACATG
FOIG_16453-NEST-F	CGTCGTTGTCGTCATTCTTCG
FOIG_16453-NEST-R	TCGCAAGGTATGTAAACTGTAATAGAAT
FOIG_16453-ID-F	ACAAACCGAGTTCTGCCGTT
FOIG_16453-ID-R	TAAACTTATGCTACACTTTGGAGGT
FOIG_16453-COM-F	CACCACCCCGGTGAACAGCTCCTCGCCCTTGCTCACATGAGGTTTCTTTGTCTCCA
FOIG_16453-COM-R	CTCGCGAAAGTCGCTGCGTTGTTTCTCTTCACTAG
FOIG_16453-Q-F	GATGGTGTCTGGGGCTTCTC
FOIG_16453-Q-R	TCGGCTCAAGGGTGTTCTTC
FOIG_16454-UP-F	AGTGGAGAAGACTGTGGTGGC
FOIG_16454-UP-R	CAAAATAGGCATTGATGTGTTGACCTCCCGTGAATGGGCCTTGGTA
FOIG_16454-DOWN-F	CTCGTCCGAGGGCAAAGGAATAGAGTAGAATGGGCGTTCTTTGGAAAAG

FOIG_16454-DOWN-R	TTGGAGAAGTCAAAAAATGGGGAG
FOIG_16454-NEST-F	GAAGGTGAAGTTGGATTGAAATTTAAAGA
FOIG_16454-NEST-R	CCTGCTCCTATATTAATTACAGAGCC
FOIG_16454-ID-F	ACCTGATGATATCACTCGTTACCC
FOIG_16454-ID-R	CTCGAGAGAATCTCGACTTCTATATGTC
FOIG_16454-COM-F	CACCACCCCGGTGAACAGCTCCTCGCCCTTGCTCACATGGCCTTAGTAAAAGTA
FOIG_16454-COM-R	CTCGCGAAAGTCGCTGCGGTGGTATGCATGTTCTTG
FOIG_16454-Q-F	TGTTCCCAGAGCCGTCAATC
FOIG_16454-Q-R	CACCAGTCGCTGACATCTGA
FOIG_16461-UP-F	ATGTTTTATTTCAGACAAGTGGGGGGC
FOIG_16461-UP-R	CAAAATAGGCATTGATGTGTTGACCTCCGGAGTATGTATAATATGTCAAAGAA
FOIG_16461-DOWN-F	CTCGTCCGAGGGCAAAGGAATAGAGTAGAGTACCAGTTCCATGGACTCT
FOIG_16461-DOWN-R	CACGAGTCATCTCTCACCACCA
FOIG_16461-NEST-F	AGGGTTTGCATCGCAACG
FOIG_16461-NEST-R	ATCTTTTTGAACAAACCCCCAGAAC
FOIG_16461-ID-F	GTCCTGTCCGTTTAAAGGATTCTCTT
FOIG_16461-ID-R	GTGCAAGCTTGTGATACCTGG
FOIG_16461-COM-F	CACCACCCCGGTGAACAGCTCCTCGCCCTTGCTCACATGCCCCATCTCTTTCCA
FOIG_16461-COM-R	CTCGCGAAAGTCGCTGCCAGATTAAAGATTTTTAG
FOIG_16461-Q-F	AGATGGCTGGCGACTTTTCT
FOIG_16461-Q-R	TGGTGACACATTCGATGCCA
MusaActin-F	TGTTGCATCCTGGTACTGCT
MusaActin-R	GGCTTTCTTGCACTGGTACAC
FocEF1α-F	GCTGGTGACTCCAAGAACGA
FocEF1α-R	CATCTTGACGATGGCGGAGT
MT-ND6-Q-F	TCCGTTTCGTTTCCCATCCC
MT-ND6-Q-R	AGCGCAAGACTTCTTCGTGA
CYTB-Q-F	TGTTCGGTGTCTCGGAGTTG
CYTB-Q-R	TGAATCGGGCGAAAACTGGA
ATP6V0C-Q-F	GTCTTCTCCTGCATGGGAGC
ATP6V0C-Q-R	GGGTTGATCCCGGTGCTTAT
HSC70-Q-F	ATTGCCGGCCTTAACGTCAT
HSC70-Q-R	GCTCTCCTGCACGAAGTGAT
sHSPs-Q-F	GCCTCAGCGACTACCTTACC
sHSPs-Q-R	GTCGATGGAGGAGGAGGTCT
SAUR-Q-F	TGTCTTGGACGCACTCACTC
SAUR-Q-R	TCCTGAAGGACCGTTGAAGC
PYR1-Q-F	CTCAAGGACGGGAGTGATGG
PYR1-Q-R	GGTGGTAACGGACCGGTAAT
FLS2-Q-F	CACCAAGAGAAGGCCTACGG
FLS2-Q-R	CTAACTCCAACACCCCCGTC

RPS2-Q-F	GGCCCGATCATCACTTCCTT
RPS2-Q-R	CCACTGAACCACTTCGCTCT
RBPs-Q-F	TGGAGGCGGTGGTTACTCTA
RBPs-Q-R	AAAGATCGGCGTCCGTAGTC

[a]UP represent PCR primers to amplify upstream fragment for the construction of targeted gene deletion mutants;
[a]UP represent PCR primers to amplify upstream fragment for the construction of targeted gene deletion mutants;
[c]NEST represent PCR primers to amplify upstream-*HPH*-downstream fragment for the construction of targeted gene deletion mutants;
[d]ID represent PCR primers for identification targeted gene deletion transformants;
[e]COM represent PCR primers to amplify full cDNA sequence of targeted genes;
[f]Q represent primers for qRT-PCR to analysis of targeted genes expression level.

Table S2 Abbreviation list in this study

Gene product	Abbreviation
NADH-ubiquinone oxidoreductase chain 6	MT-ND6
Cytochrome b	CYTB
V-type proton ATPase 16 kDa proteolipid subunit	ATP6V0C
Heat shock cognate 70 kDa protein	HSC70
Small heat shock protein	sHSPs
Auxin-induced protein 15A	SAUR
Abscisic acid receptor PYR1	PYR1
LRR receptor-like serine/threonine-protein kinase	FLS2
Disease resistance protein	RPS2
RNA-binding protein	RBPs
